# Synthesis and Properties of Ethylene/propylene and Ethylene/propylene/5-ethylidene-2-norbornene Copolymers Obtained on *Rac*-Et(2-MeInd)_2_ZrMe_2_/Isobutylaluminium Aryloxide Catalytic Systems

**DOI:** 10.3390/polym15030487

**Published:** 2023-01-17

**Authors:** Evgeny E. Faingol’d, Stanislav L. Saratovskikh, Andrei N. Panin, Olga N. Babkina, Igor V. Zharkov, Artur T. Kapasharov, Maria L. Bubnova, Gennady V. Shilov, Natalia M. Bravaya

**Affiliations:** Federal Research Center of Problems of Chemical Physics and Medicinal Chemistry, Russian Academy of Sciences, Academician Semenov Avenue 1, 142432 Chernogolovka, Moscow Region, Russia

**Keywords:** metallocene, isobutylaluminum aryloxide, catalyst, copolymerization, ethylene, propylene, 5-ethylidene-2-norbornene, EPM, EPDM, rubber, properties

## Abstract

Ethylene/propylene (E/P) and ethylene/propylene/5-ethylidene-2-norbornene (E/P/ENB) copolymers were obtained on *rac*-Et(2-MeInd)_2_ZrMe_2_ activated by a number of isobutylaluminium aryloxides: (2,6-^t^Bu_2_PhO-)Al^i^Bu_2_ (1-DTBP) (2,6-^t^Bu_2_,4-Me-PhO-)Al^i^Bu_2_ (1-BHT), (2,4,6-^t^Bu_2_PhO-)Al^i^Bu_2_ (1-TTBP), (2,6-^t^Bu_2_,4-Me-PhO-)_2_Al^i^Bu (2-BHT), (2,6-^t^Bu_2_PhO-)2Al^i^Bu (2-DTBP), [(2-Me,6-^t^Bu-C_6_H_3_O)Al^i^Bu_2_]_2_ (1-MTBP), [(2,6-Ph_2_-PhO)Al^i^Bu_2_]_2_ (1-DPP). This study shows how the structure of an activator influences catalytic activity and polymer properties, such as the copolymer composition, molecular weight characteristics, and thermophysical and mechanical properties. It has been shown that both the introduction of a bulky substituent in the para-position of the aryloxy group and the additional aryloxy group in the structure of an activator lead to a significant decrease in activity of the catalytic system in all studied copolymerization processes. Moreover, activation by bulkier aryloxides leads to lower levels of comonomer insertion and gives rise to higher molecular weight polymers. Broad or multiple endothermic peaks with different values of melting points are observed on the DSC curves of the copolymers obtained with different catalytic systems. The DSC of the thermally fractionated samples makes it possible to reveal the heterogeneity of the copolymer microstructure, which manifests itself in the presence of a set of lamellar crystallites of different thickness. The results also present the mechanical properties of the copolymers, such as the tensile strength (σ), elongation at break (ε), and engineering strain (EL). The synthesized E/P and E/P/ENB copolymers contain about 1–4 wt.% of the sterically hindered phenols obtained in situ as a residue of the hydrolyzed activators in the course of reaction quenching. This determines the increased thermooxidative stability of the copolymers.

## 1. Introduction

Ethylene/propylene and ethylene/propylene-diene rubbers (also called as EPM and EPDM) are important elastomeric polymers widely used in cars, cables, construction, and other industries [1,2,3,4,5]. These elastomeric materials are used as materials showing excellent resistance to heat, air, ozone, and steam, and are able to operate in a wide temperature range (from −50 to +130 °C). EPDM can be functionalized or vulcanized via the diene moieties. EPDM may be compounded with different fillers such as carbon black, and with plasticizers such as paraffinic oils. Application areas and demand for EP(D)M still expand, and the forecast of annual production growth is 7.2% [5].

The production of ethylene copolymers and EPDM is based solely on the catalytic coordination polymerization processes. Catalytic systems consist of a precatalyst and an activator. The type of catalytic system affects key properties of polymer products such as the microstructure, crystallinity, glass transition temperature, molecular weight characteristics, thermal, and mechanical quality. [1,2,6,7]. The most effective and modern precatalysts are metallocenes and post-metallocenes activated by methylalumoxane (MAO). Notable species are metallocenes [2,3,8,9,10,11,12], constrained geometry complexes [2,3,13,14,15], titanium half-sandwiches with donor nitrogen-containing ligands [2,3,4,13,16], and aryloxy-ether zirconium or hafnium complexes [2,3,17]. Some precatalysts have become the basis of novel technologies such as Keltan ACE and AMC (Advanced Molecular Catalyst) [2,3,16,17]. The discovery of new suitable complexes and the novel means of their activation could lead to new and possibly much cheaper processes for the production of EPDM with a wide spectrum of properties [2,3].

Our previous work shows that isobutylaluminum aryloxides work well as activators of dimethyl bis-indenyl metallocene complexes in olefin (co)polymerization [18,19,20,21]. New catalytic systems have been found to be effective for producing ethylene/propylene copolymers and ethylene/propylene/5-ethylidene-2-norbornene terpolymers. It was shown that the structure of the metallocene precatalyst strongly influences the copolymer composition, molecular weight, and thermophysical and mechanical properties [21]. On the other hand, the differences in aryloxide structures should also affect catalytic properties and properties of polymer products. In this work, we show how the different aryloxide activators used in ethylene/propylene and ethylene/propylene/5-ethylidene-2-norbornene copolymerization reactions change the activity of catalytic systems, the composition of copolymers, the molecular weight characteristics, and the thermo physical and mechanical properties of the copolymers using one precatalyst *rac*-Et(2-MeInd)_2_ZrMe_2_.

## 2. Materials and Methods

### 2.1. Reagents

High purity toluene was distilled over LiAlH_4_ and kept over molecular sieves (4 A) in argon atmosphere (99.999% purity). *rac*-EtInd_2_ZrMe_2_ was synthesized by alkylation of corresponding metallocene dichloride (Boulder Scientific) according to the standard technique [22]. Isobutylaluminum aryloxides (2,6-^t^Bu_2_PhO-)Al^i^Bu_2_ (1-DTBP) (2,6-^t^Bu_2_,4-Me-PhO-)Al^i^Bu_2_ (1-BHT), (2,4,6-^t^Bu_2_PhO-)Al^i^Bu_2_ (1-TTBP), (2,6-^t^Bu_2_,4-Me-PhO-)_2_Al^i^Bu (2-BHT), (2,6-^t^Bu_2_PhO-)_2_Al^i^Bu (2-DTBP), [(2-Me,6-^t^Bu-C_6_H_3_O)Al^i^Bu_2_]_2_ (1-MTBP), [(2,6-Ph_2_-PhO)Al^i^Bu_2_]_2_ (1-DPP) were obtained by reaction of Al^i^Bu_3_ (Aldrich Chem. Co., Milwaukee, MI, USA) with the corresponding phenol according to the procedures described in our works [19,20,21]. All manipulations with *rac*-Et(2-MeInd)_2_ZrMe_2_ and isobutylaluminum aryloxides were performed in an argon atmosphere.

### 2.2. Copolymerization of Ethylene and Propylene

Polymerization was carried out in a 200 mL stainless steel reactor equipped with a mechanical stirrer and a temperature-controlled jacket. The reactor was vacuumed for 1 h at 50 °C, filled with argon and cooled down to room temperature. The ampoule with *rac-*Et(2-MeInd)_2_ZrMe_2_ was placed in the reactor in an argon counter flow. The reactor was evacuated and then charged with an activator dissolved in toluene of desired concentration providing an Al/M = 300 molar ratio. The reactor was heated to 30 °C, and the reaction mixture was saturated with a mixture of ethylene and propylene at a desired molar ratio. Polymerization was initiated by a catalyst introduced into reaction medium by breaking the ampoule with metallocene inside the reactor.

Total ethylene/propylene pressure was maintained constant through all polymerization process. The process was stopped by adding 5% solution of hydrochloric acid in ethanol. The polymer was washed in a water/ethanol mixture, recovered by filtration and dried in vacuum at 60 °C. 

The E/P copolymer obtained on the *rac*-Et(2-MeInd)_2_ZrMe_2_/isobutylaluminumoxane (IBAO) catalyst system was used as a reference sample for comparison of the resistance of aryloxide copolymers to thermal oxidative. IBAO was synthesized by controlled hydrolysis on ice according to the procedure described in [23,24]. E/P copolymer characteristics: Mw = 115,000, Mw/Mn = 3.1, E/P composition = 91/9 mol/mol.

### 2.3. Terpolymerization of Ethylene, Propylene and 5-Ethylidene-2-norbornene

We used the same method for terpolymerization of ethylene, propylene, and 5-ethylidene-2-norbornene. The only difference is that the toluene solution of 5-ethylidene-2-norbornene was added together with a solution of an activator.

### 2.4. Polymer Analysis

Molecular weight and molecular weight distribution of copolymers as solutions in 1,2,4-trichlorobenzene were determined on a high-temperature PL-220 liquid gel-chromatograph with PL Gel Olexis columns and a refractometer detector at 135 °C. The flow rate was 1 cm^3^/min. Calibration curves were based on polyethylene and polystyrene standards.

Differential scanning calorimetry (DSC) analysis was performed using a DSC 822 (Metler Toledo, Schwerzenbach, Switzerland) device with STARe 15.0 software with the scanning rate of 10 degrees/min under inert atmosphere in the −100–170 °C temperature range. Heat of fusion and melting temperatures were determined at the second heating cycle.

Thermal fractionation of polymer samples (in DSC crucibles) was performed as follows. The thermal history of the samples was removed by heating the polymer samples to 140 °C and keeping them at this temperature for 10 min. The temperature was then decreased in 10 °C steps to room temperature. The samples were left isothermal for 220 min after each step. A step cooling rate of 5°/min was used. Afterwards, DSC thermograms of fractionated samples were recorded.

Simultaneous evaluation of mass changes (TGA) and thermal effects occurring in the samples (DSC) was performed using a TGA/SDTA851e calorimeter (Metler Toledo, Schwerzenbach, Switzerland) in the 25–500 °C range at a scanning rate of 10 °C/min in air atmosphere.

X-ray diffraction patterns of copolymer films were obtained on an ARL X’TRA diffractometer (THERMOFISHER SCIENTIFIC, Ecublens, Switzerland) equipped with a solid-state detector using Θ−Θ geometry in the 5–50° range with a scan step of 0.02°; the exposure time per frame was 1 s.

Monomer content in copolymers and the residual content of phenols in the samples were determined by FTIR spectroscopy of polymer films according to the methods described in [25,26,27,28]. IR spectra were recorded on a Perkin–Elmer Spectrum 100 spectrometer (Shelton, CT, USA).

### 2.5. Dynamic Mechanical Analysis

Dynamic mechanical properties were measured using a Netzsch DMA 242 C dynamic mechanical analyzer in a uniaxial stretching mode on film samples of rectangular shape (10 × 3 × 0.3 mm) at continuous increase in temperature from –170 to 130 °C with a rate of 2 °C/min in helium atmosphere. Sinusoidal oscillating stress applied to the samples allowed one to develop the 30 μm deformation amplitude at 1 Hz frequency. Glass transition temperature (Tg) was determined from the temperature dependencies either by the onset point of elastic modulus curves (E′) or by maximum position of the loss modulus curves (E″).

### 2.6. Polymer Mechanical Tests

Mechanical tests of copolymers were carried out on a 2166 P-5 stretch-breaking machine at room temperature according to the standard ISO 37 technique (Die type 1). The results were averaged out of five samples. The stretching speed was 10 mm/min. Engineering strain (EL) of the copolymers was determined by the formula: EL = (l_1_ − l_0_)/l_0_ × 100, where l_1_ is the length of the working section along with two folded parts of the broken sample, and l_0_ is the initial length of the working section.

### 2.7. DFT Calculations

DFT calculations were performed using the Gaussian 09 package, employing functional wB97x-D, which was also used previously [20,29]. Molecules involved are of considerable size to fit into computationally feasible ~1000 basis functions budget, and basis sets used are 6-311+G(d,p) for Al and O atoms, and 6-31G(d) for C and H atoms. SCRF (PCM) model was added to account for solvent (toluene) effect.

## 3. Results and Discussions

**Figure 1:**
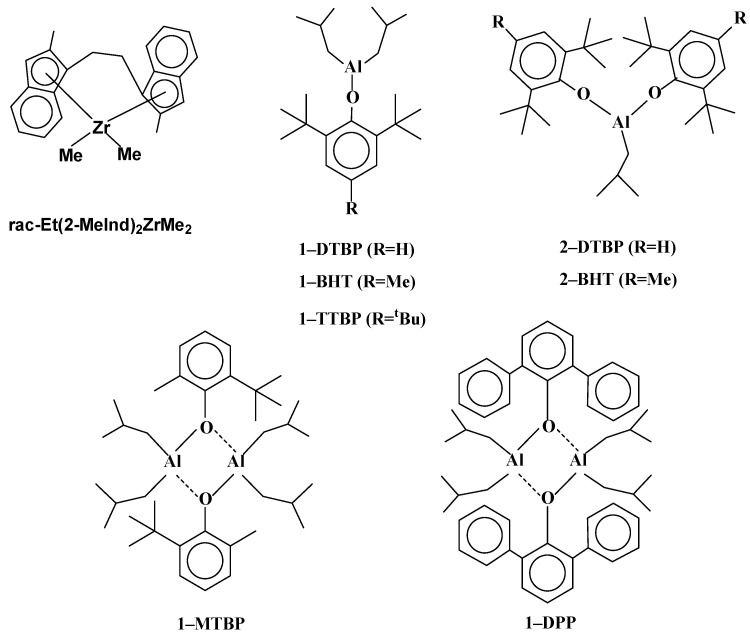
*rac*-Et(2-MeInd)_2_ZrMe_2_ and isobuthylaluminum aryloxides used in the study.

Sterically hindered isobuthylaluminum aryloxides with ortho-^t^Bu substituted aryloxy ligands efficiently activate the dimethylated bis-indenyl catalysts in the homo- and co-polymerization of olefins and the terpolymerization with 5-ethylidene-2-norbornene [17,18,19,20]. The structure of the organoaluminum species as the cocatalyst can significantly influence the catalytic properties due to steric reasons at the least. To study this effect, we have used *rac*-Et(2-MeInd)_2_ZrMe_2_ as a precatalyst and a number of mono- and di-aryloxide activators shown in Figure 1. 1-MTBP and 1-DPP are shown as dimers since their dimer structure was identified in solid state (X-ray) [20]. Previously, this zirconium precatalyst was shown to be the most active among several bridged bis-indenyl metallocenes in the homo- and copolymerization of olefins under activation with all indicated aryloxides [21].

### 3.1. Copolymerization Results

The results of E/P and E/P/ENB copolymerization with *rac*-Et(2-MeInd)_2_ZrMe_2_ and different activators and the properties of the obtained copolymers are presented in Table 1.

Table 1 shows that the structure of the aluminum aryloxide activator significantly influences the catalytic activity of the system, the molecular mass and the composition of the copolymers. Unexpectedly, the substituent in the para-position of the aryloxy ligand of the activator has a significant effect on the catalytic properties of the system. One can see that the increase in substituent volume (H→Me→^t^Bu) leads to a decrease in catalytic activity from 92.4 to 74.4 tons copolym./(mol Zr 3 min) in E/P copolymerization, an increase in Mw of the copolymer from 89,000 to 143,000, and a decrease in the propylene content in the copolymer from 14–15 to 9 mol % (entries 1–3). The introduction of a diene comonomer is usually accompanied by a significant decrease in catalytic activity [30,31]. The dependencies of the listed characteristics on the type of activator are less pronounced but also seen in ternary copolymerization (entries 8–10).

**Figure 2:**
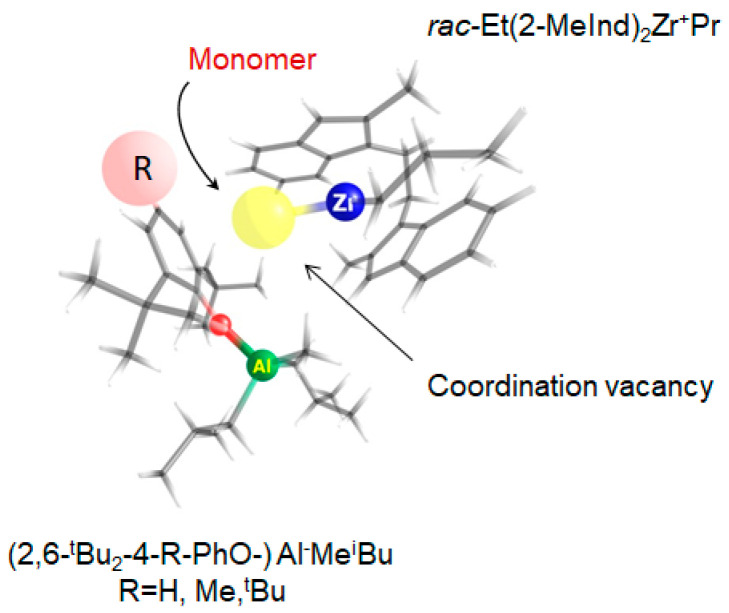
DFT optimized structure of catalytically active center *rac*-Et(2-MeInd)_2_Zr^+^Pr…(2,6-^t^Bu_2_-4-R-PhO-)Al^–^Me^i^Bu, where R is *para*-substituent in the aryloxy fragment.

The decrease in copolymer incorporation, in particular, indicates the relevance of steric hindrance in *para-*substituent of the aryloxy group in the cocatalyst. This can be rationalized by a model in which, after the initial displacement by an incoming monomer, the anion stays fairly close to the cationic active center. For such a case, several conformations were computationally (DFT) analyzed, with -CH2-CH2-CH3 representing the growing chain. Al–Zr distances are in the range 5.5–6 Ǻ. Judging by the energies (ωB97XD/6-31+G**), the most favored is the conformation where it is the aryloxy group that is the closest one to the monomer insertion site. In such an arrangement, as illustrated on Figure 2, the *para-*substituent is in the vicinity of the possible monomer approach trajectory. This speculative model also relies on the fact that the bridged bis-indenyl ligand already limits the steric space where it is feasible for the monomer to approach.

The introduction of the additional aryloxy ligand in the structure of the activator leads to a ~2.5-fold drop of the activity in the E/P copolymerization (entries 1,4 and 2,5) and a 4-fold drop in the E/P/ENB terpolymerization (entries 8,11 and 9,12). The propylene content in the copolymers obtained with the di-aryloxides is lower than that with the monoaryloxides (11% and 8% for E/P, entries 4,5; 7% and 5% for E/P/ENB, entries 11,12). However, the molecular weights of the terpolymers obtained with the di-aryloxide activators are much higher than those with the monoaryloxides (17,100 vs. 10,800, entries 11, 8 and 181,000 vs.105,000 entries 12, 9).

*Ortho*-substituents of aryloxy ligands are also key structural elements of the aryloxide activator. It was shown that the substituents smaller than ^t^Bu enable the dimerization of the respective aryloxides [20]. Among a number of such species, only two aryloxides (1-MTBP and 1-DPP) are least stable in solution as dimers and are able to activate zirconocene. In regard to the activity in the E/P copolymerization, the molecular weight of copolymers, and the copolymer composition with 1-MTBP as the activator is close to 1-DTBP, and 1-DPP is between the mono- and di-aryloxides (Table 1, entries 6, 7). In the E/P/ENB terpolymerization (entries 13, 14), activity of the system with 1-MTBP is also between those of the mono- and di-aryloxides and is about 2 times lower than in the E/P copolymerization. 1-DPP provides the lowest activity, which can be due to lower degree of monomerization as compared with 1-MTBP.

### 3.2. Molecular Weight Characteristics, Crystallinity, and Thermal Properties of Copolymers 

The molecular weights of the E/P and E/P/ENB copolymers are higher for the copolymers obtained with bulkier activators and are in the range from 89,000 to 181,000 with Mw/Mn = 2–4 (Table 1). As indicated above, this correlation can be due to the lower probability of the chain transfer in the presence of the bulkier activator. 

**Figure 3:**
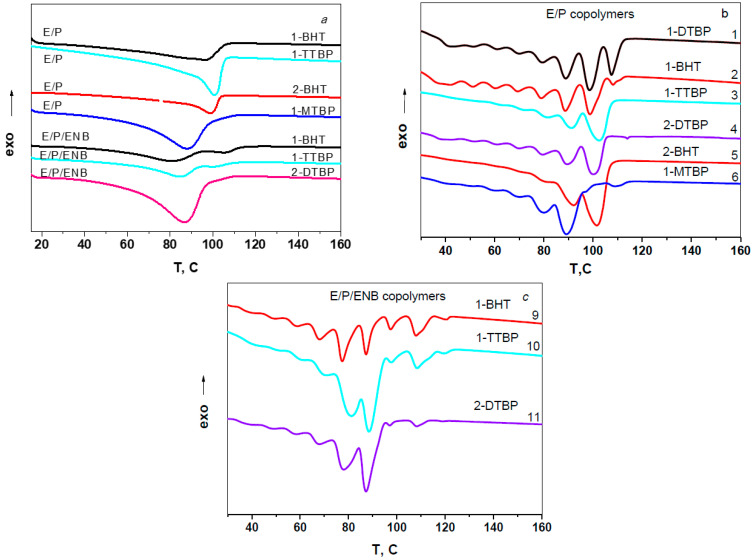
DSC thermograms for second cycle heating of pristine (**a**) and fractionated (**b**,**c**) E/P and E/P/ENB copolymers obtained on the *rac*-Et(2-MeInd)_2_ZrMe_2_/isobuthylaluminum aryloxide catalyst systems.

All E/P copolymers show wide asymmetrical melting peaks close to 100 °C on the DSC thermograms at the second cycle heating of the samples, while the DSC curves for the E/P/ENB copolymers are polymodal (Figure 3a). The polymodality and peak widening indicate the compositional inhomogeneity of copolymers.

The crystallinity values of the terpolymers defined by both DSC and X-ray data (columns ΔH and χ in Table 1) are much lower than those for the E/P copolymers even at a lower molar fraction of the propylene content and only 2–4 mol % of the diene moieties. This is probably due to the possibility of the propylene units’ inclusion into the PE crystallites in the case of the E/P copolymers [32,33,34], and the impossibility of such inclusions for the bulky branches is due to their larger size. In addition, the presence of the bulky ENB units in the copolymer reduces the chain mobility during crystallization. Thus, the sequences containing the ENB units should be present only in the amorphous phase of the copolymers leading to the lower crystallinity values for the terpolymers.

Stepwise isothermal crystallization from polymer melt is a useful technique for the evaluation of the chain heterogeneity in such copolymers [35,36,37,38]. The DSC curves of thermally fractionated copolymers are presented in Figure 3b,c.

For the fractionated copolymers, the presence of several melting peaks in a wide temperature range of stepwise crystallized copolymers (Figure 3b,c) points out to the presence of lamellar crystallites of different thickness. The higher values of T_m_ correspond to the larger lamellae thickness due to the intra- and intermolecular chain crystallization with the longer methylene sequences. The number of endothermic peaks of the fractionated samples and their distributionin intensity suggests the manner by which the comonomers are distributed in the macromolecular chains. A smaller number of peaks and a greater difference in their intensity should indicate a more uniform microstructure of the copolymers in terms of the length of the methylene sequences.

Table 2 lists the integral intensities of the three more intensive peaks related to that of the most intense one for the co- and terpolymers, the melting points at which they are observed (in parentheses), and their summarized percentage contribution to the total integral intensity of the peaks. One can see that the melting points of the more intensive peaks of the E/P copolymers are much higher than those for the terpolymers at about a similar or even higher molar fraction of the comonomer(s) in the copolymers. The differences in the relative intensity of the main melting peaks for both the co- and terpolymers also reflect the specifics of the catalytic systems with the different activators. The increase in the steric volume of the *para*-substituent in the aryloxide activator (from 1-DTBP to 1-TTBP) is accompanied by the decrease in the catalyst activity, the comonomer content in E/P copolymers (Table 1), and the number of high melting fractions with longer methylene sequences (Figure 3b, Table 2). The triads of the most intense peaks are shifted by approximately 10 °C toward the lower temperatures. Therefore, it is probable that the coordination of the bulkier propylene comonomer prior to its incorporation temporarily blocks the active site for ethylene, thus limiting the formation of the extended ethylene sequences (see Figure 2). Similar effects are also observed upon going from monosubstituted to much more steric disubstituted aryloxides.

### 3.3. DMA Analysis of Copolymers

**Figure 4:**
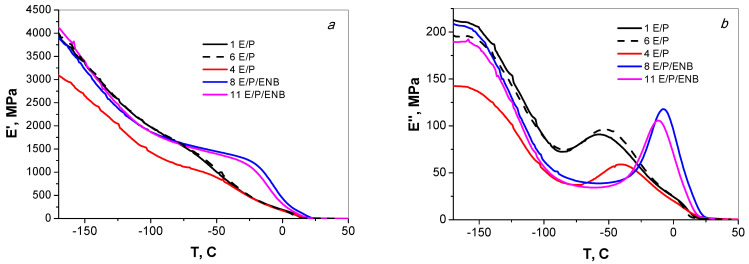
Temperature dependencies (at 1 Hz) of E′ (**a**) and E″ (**b**) for E/P copolymers obtained on catalytic systems with mono- and dimeric aryl oxides (1) 1-DTBP, (6) 1-MTBP, (4) 2-DTBP, and E/P/ENB terpolymers obtained on catalytic systems with (8) 1-DTBP and (11) 2-DTBP. Numbers on the curves match the entries in Table 1 and Table 3.

DMA was used to compare the mechanical behavior of the copolymers obtained with the 1-DTBP, 1-MTBP, and 2-DTBP activators. Figure 4 shows the temperature dependencies of the storage modulus (E′) and the loss modulus (E″) of the samples. It should be noted that during the DMA tests, all the samples retained the shapeand did not flow up to temperatures approaching the melting points of the samples. The parameters obtained from the DMA are listed in Table 3. One can see that in the range from −70 °C to 20 °C, E/P/ENB terpolymers obtained with 1-DTBP (curve 8) and 2-DTBP (curve 11) show the higher E′ values than the E/P copolymers (curves 1,6,4). Probably, this is because bulky ENB units do not fit the crystalline phase and are contained to the amorphous phase instead, restricting mobility of the chains in it, which in turn contributes to the increased rigidity of the material. The E/P copolymers formed with 1-DTBP (curve 1) and 1-MTBP (curve 6) show almost the identical behavior of the temperature dependencies of E′ and coincide with the curves for the terpolymers at temperatures above 20 °C. The storage modulus for the E/P copolymer obtained with diaryloxide (curve 4) is lower in a the low-temperature range up to −40 °C compared to the E/P copolymers obtained with the monoaryloxides, thus showing the lower rigidity of the copolymer. The glass transition temperatures determined by a set of the E′ temperature curves (curves 1,6) are close in values (−51, −49 °C, correspondingly), and Tg is about 10 degrees higher (−38 °C) for the E/P copolymer obtained with diaryloxide. In the E/P/ENB terpolymers, the presence of the diene monomer significantly raises Tg, namely, to −11–−8 °C.

The temperature dependencies of the loss moduli E″ for the E/P and E/P/ENB copolymers obtained with the monoaryloxide activators are almost identical in pairs (curves 1,6; 8,11, correspondingly) but differ significantly in the position of the peak maxima. The T_g_ values of the copolymers determined by the E″ maxima are very close to those determined from the E′ curves. Thus, the E/P/ENB terpolymers are more rigid than the E/P copolymers and thus show the lower ability of the materials to dissipate energy.

### 3.4. Mechanical Analysis of Copolymers

**Figure 5:**
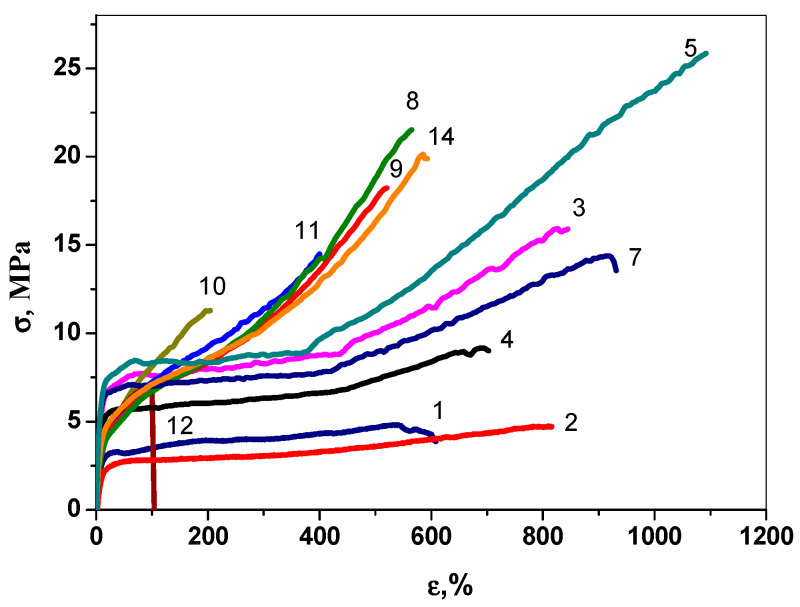
Stress–strain diagrams for E/P samples 1–5, 7 and E/P/ENB samples 8–12, 14, produced by *rac*-Et(2-MeInd)_2_ZrMe_2_/isobutylaluminum aryloxide catalytic systems. Aryloxides are 1, 8—1-DTBP; 2, 9—1-BHT, 3, 10—1-TTBP; 7, 14—1-DPP; 4, 11—2-DTBP, 9, 12—2-BHT.

The stress–strain diagrams of the E/P copolymers (Figure 5) are typical for thermoplastic materials showing the presence of yield points and rubbery plateau (curves 1–5,7). The E/P/ENB terpolymers show elastomeric behavior without yield points (curves 8,10–12,14). The pronounced strain hardening (at about 400% of strain) is observed for the E/P copolymers obtained with the most sterically bulky 1-TTBP, 2-DTBP, and 2-BHT activators, which provide the formation of copolymers with a lower comonomer content and, correspondingly, a higher crystallinity (column ΔH in Table 1). The strain-hardening effect is mainly determined by the disentanglement and alignment of the chains between the crystallites, and the recrystallization of the polymer chains as a result of deformation. Samples 3 and 5 show that the more uniform distribution of temperature revealed fractions and at the same time the best mechanical properties (σ_r_ = 25 MPa, ε_r_ = 1050%, EL = 820%). Monomeric 1-MTBP produces the E/P copolymer with the worst mechanical properties (σ = 3MPa, ε = 290%). The terpolymers obtained with the bulkier activators show lower values of σ, ε and EL (compare entries 8,10 and 9,12 in Table 1 and data in Figure 5). On the whole, the tensile strength of the terpolymers is higher than that of the copolymers. At the same time, the higher compositional inhomogeneity of the ternary copolymers should be noted, as seen by the double and triple melting peaks in six of the seven tested samples (Table 1.) The inhomogeneity aspect can also contribute to the mechanical properties. The terpolymer produced with dimeric 1-DPP shows the best mechanical characteristics.

### 3.5. Thermooxidative Destruction of Copolymers

**Figure 6:**
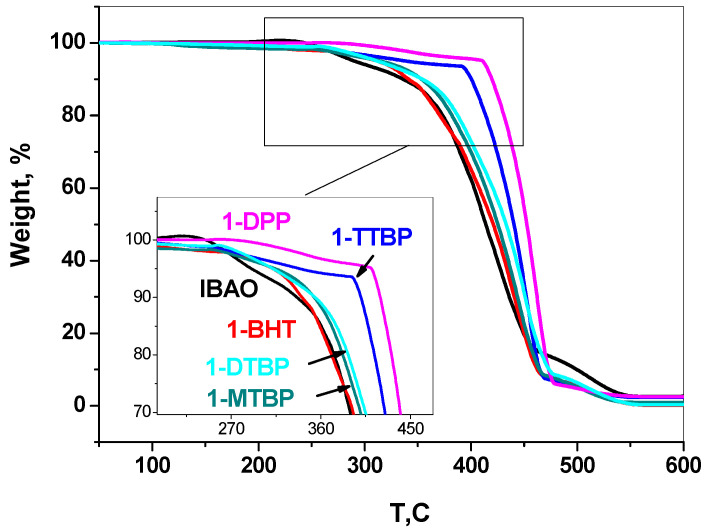
The effect of phenol antioxidant on thermooxidative destruction of E/P copolymers. TGA of polymers produced by *rac*-Et(2-MeInd)_2_ZrMe_2_/activator catalytic system, activator = IBAO (isobutylalumoxane), 1-DTBP, 1-BHT, 1-TTBP, 1-MTBP, 1-DPP.

Sterically hindered phenols such as ionol and irganox 1010 are conventional stabilizers for polyolefins [39,40]. They prevent thermooxidative destruction and extend the service life of polymeric materials. The isobuthylaluminum aryloxides used by us for activation are hydrolyzed after the reaction quenching to generate sterically hindered phenols structurally similar to ionol. The phenol residue in the washed polymers amounts to 1–4 wt.%. The E/P TGA thermograms in the air atmosphere show a higher thermooxidative stability of the E/P copolymers as compared to the copolymer obtained in the case of activation by phenol-free isobuthylalumoxane (IBAO) (see Experimental) (Figure 6, Table 4). The best thermooxidative stability is observed for the samples obtained with 1-TTBP and 1-DPP. Moreover, 50% weight loss of these samples is observed at temperatures by 45 and 52 degrees higher than for an unstabilized sample. The higher stabilizing effect of these two phenols can be explained by their superior ability to terminate radical chain decomposition via interaction with the polymer radicals and/or the formation of radicals with delocalized electron density [40].

## 4. Conclusions

The study shows that the structure of the isobuthylaluminum aryloxide activator influences the activity of the catalytic system based on *rac*-Et(2-MeInd)_2_ZrMe_2_ in E/P and E/P/ENB copolymerization, and the composition and molecular masses of the copolymers, which in turn affect the thermophysical and mechanical properties. The DFT calculations show that the main reason of the effects consists in the most energetically favorable specific coordination of the activator to the zirconium active center by the aryl oxide group. The presence of the bulky substituents in the para-position of the aryloxide fragment in the vicinity of the zirconium center prevents the approach and incorporation of the bulky comonomers into the active center (lower activity and lower content of comonomer in copolymers) and restricts the chain transfer reactions (higher molecular weight of copolymers). The crystallinity of the E/P copolymers (DSC and X-ray data) is considerably higher than that of the E/P/ENB copolymers at a considerably lower comonomer content in the latter. The DSC analysis of the thermally fractionated copolymers shows a non-uniform distribution of comonomers in the copolymers showing the presence of multiple DSC peaks. These sets of DSC peaks originate from the presence of sets of lamellar crystallites of different thickness in the copolymers formed by the inter- or intramolecular crystallization of the methylene sequences of different lengths. Analysis of the strain–stress diagrams reveals that all the E/P copolymers show thermoplastic behavior, while the terpolymers behave as elastomers under stress. The E/P copolymer obtained with 2-BHT shows the best mechanical properties (σ_ρ_ = 25 MPa, ε_ρ_ = 1150%, EL = 820%). The best mechanical properties among the terpolymers are demonstrated by the copolymer obtained with 1-DPP (σ_ρ_ = 20 MPa, ε_ρ_ = 870%, EL = 320%). The copolymers contain residual phenol antioxidants and their content is found to be in the 1–4 wt.% range. This is a product of catalytic system decomposition during the reaction quenching and polymer washing. The samples show a noticeable increase in thermooxidative stability as compared to phenol-free samples.

## Figures and Tables

**Table 1:** Copolymerization of ethylene with propylene and terpolymerization of ethylene, propylene and 5-ethylidene-2-norbornene on *rac*-Et(2-MeInd)_2_ZrMe_2_/isobutylaluminum aryloxide, copolymer properties.

№	Activator	M *^a^*^)^	A *^b^*^)^	M_w_	M_w_/M_n_	Copolymer Composition E/P/ENB mol. %	T_m_ *^c^*^)^, °C	∆H *^d)^*, J/g	χ *^e^*^)^, % X-ray	σ_r_ *^f^*^)^, MPa	ε*_r_^g^*^)^, %	EL *^h^*^)^ %
1	1-DTBP	E/P	92.4	89,000	2.7	86/14/-	101	71.5	39	5.0	600	130
2	1-BHT	E/P	79.6	108,000	2.7	85/15/-	96	59.9	33	5.2	850	190
3 *^k^*^)^	1-TTBP	E/P	74.4	143,000	2.3	91/9/-	102	88.6	37	15.8	840	610
4 *^k)^*	2-DTBP	E/P	36.6	96,000	2.2	89/11/-	99	77.0	36	9.0	700	500
5 *^k)^*	2-BHT	E/P	32.8	160,000	2.3	92/8/-	101	95.2	42	25.0	1050	820
6 *^l)^*	1-MTBP (dimer)	E/P	85.8	98,000	3.5	86/14/-	102	68.8	35	3.0	290	-
7 *^l)^*	1-DPP (dimer)	E/P	48.6	105,000	2.5	88/12/-	103	70.9	42	13.4	900	740
8 *^k)^*	1-DTBP	E/P/ENB	39.2	111,000	2.6	85/13/2	85, 101	58.8	21	21.6	560	210
9 *^k)^*	1-BHT	E/P/ENB	35.2	115,000	3.0	88/8/4	81, 106	57.7	21	18.0	520	230
10 *^k)^*	1-TTBP	E/P/ENB	32.2	n.s.*^i^*^)^	-	90/8/2	85, 101, 120	56.7	24	11.0	200	130
11 *^k)^*	2-DTBP	E/P/ENB	9.2	176,000	2.3	91/7/2	87	56.7	27	14.0	400	170
12 *^k)^*	2-BHT	E/P/ENB	8.6	181,000	2.2	93/5/2	83, 99, 122	57.0	27	7.0 *^k)^*	100 *^j)^*	-
13 *^l)^*	1-MTBP (dimer)	E/P/ENB	19.6	165,000	2.2	91/7/2	90, 105	64.3	-	13.7	290	210
14 *^l)^*	1-DPP (dimer)	E/P/ENB	7.6	n.s.*^i^*^)^	-	91/6/3	44, 86, 98	55.1	32	19.6	570	320

Copolymerization conditions: [Zr] ≈ 9 × 10^−5^ mol/l, Al/M = 300 mol/mol, monomer pressure—10.6 atm, toluene—60mL., 30 °C; *^a)^* Monomers: E—ethylene, P—propylene, ENB—5-ethylidene-2-norbornene;E/P = 0.7/1, E/P/ENB = 4.7/4.3/1; *^b^*^)^ Specific activity of the system determined by consumption of gaseous monomer in 3 min. of reaction time and related to the amount of catalyst and atmosphere in ton copolymer/(mol M × 3 min × atm); *^c^*^)^ Melting point of the copolymers determined by the maximum of DSC curve; *^d^*^)^ Heat of fusion; *^e^*^)^ Crystallinity determined from X-ray powder diffraction analysis; *^f^*^)^ Tensile strength; *^g^*^)^ Elongation at break; *^h^*^)^ Engineering strain; *^i^*^)^ Copolymer is not soluble in 1,2,4-trichlorobenzene at 135 C; *^j)^* Mechanical tests are performed on four blades; *^k^*^)^ M_w_, M_w_/M_n_, copolymer composition and T_m_ are from reference [19]; *^l^*^)^ M_w_, M_w_/M_n_, copolymer composition and T_m_ are from reference [20].

**Table 2:** Results of thermal fractionation of E/P and E/P/ENB copolymer samples.

№ *^a^*^)^	E/P Content Mol. % *^b^*^)^	Activator	Peak Ratio *^c^*^)^	∑ of Three Peaks, *^d^*^)^ %
			1 Peak/2 Peak/3 Peak (T_m_)	
1	14/0	1-DTBP	0.75(79)/0.90 (89)/1.00 (99)	63
2	15/0	1-BHT	0.79 (79)/1.00 (89)/0.76 (99)	48
3	9/0	1-TTBP	0.50 (82)/0.66 (91)/1.00 (101)	74
4	11/0	2-DTBP	0.64 (80)/0.86 (90)/1.00 (100)	64
5	8/0	2-BHT	0.40 (81)/0.68 (92)/1.00 (101)	75
6	14/0	1-MTBP	0.35 (70)/0.36 (80)/1.00 (90)	66
9	8/4	1-BHT	0.78 (69)/1.00 (78)/0.65 (87)	57
10	8/2	1-TTBP	0.58 (70)/1.00 (81)/0.96 (89)	66
11	7/2	2-DTBP	0.57 (68)/0.99 (78)/1.00 (87)	74 *^e)^*

*^a^*^)^ Numbers on the curves match the entries in Table 1; *^b^*^)^ Molar fraction of propylene (P) and 5-ethylidene-2-norbornene (ENB) in copolymer; *^c^*^)^ Ratios of the three most intense peaks (melting points are given in parentheses), Integral intensity of the maximum peak is taken as a unit; *^d^*^)^ Total share of the square of three most intense peaks from total square of all observable peaks; *^e^*^)^ The intensity of the second and third peaks is 58% of all melting peaks.

**Table 3:** DMA data for E/P and E/P/ENB copolymers.

№ *	Activator	Copolymer	E′ (−70 °C), MPa	E′ (0 °C), MPa	PeakE″, MPa	T_g_ (E′), °C	T_g_ (E″), °C
1	1-DTBP	E/P	1469	190	91	−51	−57
6	1-MTBP	E/P	1495	190	95	−49	−53
4	2-DTBP	E/P	1095	190	59	−38	−40
8	1-DTBP	E/P/ENB	1537	311	106	−8	−8
11	2-DTBP	E/P/ENB	1575	433	118	−11	−11

* Numbers on the curves match the entries in Table 1.

**Table 4:** Temperature values of mass loss in air in the range from 5 to 50% for E/P copolymers obtained with different monoaryloxide activators.

Activator	T (∆m = 5%), °C	T (∆m = 10%), °C	T (∆m = 50%), °C
IBAO	290	335	411
1-BHT	313	340	436
1-MTBP	316	353	449
1-DTBP	313	355	453
1-TTBP	340	400	456
1-DPP	410	419	467
